# A Mouse Model of Visual Perceptual Learning Reveals Alterations in Neuronal Coding and Dendritic Spine Density in the Visual Cortex

**DOI:** 10.3389/fnbeh.2016.00042

**Published:** 2016-03-10

**Authors:** Yan Wang, Wei Wu, Xian Zhang, Xu Hu, Yue Li, Shihao Lou, Xiao Ma, Xu An, Hui Liu, Jing Peng, Danyi Ma, Yifeng Zhou, Yupeng Yang

**Affiliations:** Chinese Academy of Sciences Key Laboratory of Brain Function and Diseases, School of Life Sciences, University of Science and Technology of ChinaHefei, China

**Keywords:** mouse behavior, discrimination learning, mouse models, contrast sensitivity, visual acuity, mouse primary visual cortex, visual water task, visual perceptual learning

## Abstract

Visual perceptual learning (VPL) can improve spatial vision in normally sighted and visually impaired individuals. Although previous studies of humans and large animals have explored the neural basis of VPL, elucidation of the underlying cellular and molecular mechanisms remains a challenge. Owing to the advantages of molecular genetic and optogenetic manipulations, the mouse is a promising model for providing a mechanistic understanding of VPL. Here, we thoroughly evaluated the effects and properties of VPL on spatial vision in C57BL/6J mice using a two-alternative, forced-choice visual water task. Briefly, the mice underwent prolonged training at near the individual threshold of contrast or spatial frequency (SF) for pattern discrimination or visual detection for 35 consecutive days. Following training, the contrast-threshold trained mice showed an 87% improvement in contrast sensitivity (CS) and a 55% gain in visual acuity (VA). Similarly, the SF-threshold trained mice exhibited comparable and long-lasting improvements in VA and significant gains in CS over a wide range of SFs. Furthermore, learning largely transferred across eyes and stimulus orientations. Interestingly, learning could transfer from a pattern discrimination task to a visual detection task, but not vice versa. We validated that this VPL fully restored VA in adult amblyopic mice and old mice. Taken together, these data indicate that mice, as a species, exhibit reliable VPL. Intrinsic signal optical imaging revealed that mice with perceptual training had higher cut-off SFs in primary visual cortex (V1) than those without perceptual training. Moreover, perceptual training induced an increase in the dendritic spine density in layer 2/3 pyramidal neurons of V1. These results indicated functional and structural alterations in V1 during VPL. Overall, our VPL mouse model will provide a platform for investigating the neurobiological basis of VPL.

## Introduction

Visual perceptual learning (VPL) refers to significant and long-lasting improvements in visual performance resulting from repetitive training on a visual task such as grating orientation discrimination (Goldstone, 1998; Gilbert et al., 2001; Lu et al., 2011). Visual performance usually includes contrast sensitivity (CS) (Li et al., 2009; Zhou et al., 2012), visual acuity (VA) (Astle et al., 2010; Yehezkel et al., 2015) and Vernier acuity (Poggio et al., 1992; Levi et al., 1997). Recently, VPL has been considered one of the most promising rehabilitative approaches for visually impaired populations, especially adult amblyopia (Polat et al., 2004; Levi, 2005; Zhou et al., 2006). However, full recovery of degraded VA in adult amblyopic patients using VPL remains a challenge. Thus, a better understanding of the mechanisms underlying VPL is important for developing more effective treatments for adult amblyopia.

Experimental animal models are important for understanding the neurobiological basis of VPL and for the development of therapeutic interventions. Much of the current understanding of the neural mechanisms underlying VPL is derived from studies of humans and large mammalian models, such as monkeys and cats. Researchers still debate the extent to which perceptual improvement stems from the early visual cortex compared to the higher-level visual cortex and decision-making areas (Schoups et al., 2001; Li et al., 2004; Yang and Maunsell, 2004; Law and Gold, 2008). More importantly, elucidation of the cellular and molecular mechanisms underlying VPL remains challenging.

In contrast, rodents provide a tremendous degree of experimental flexibility through the use of many cutting-edge tools, such as transgenics, optogenetics, *in vivo* whole-cell recording and two-photon imaging. Moreover, the mouse is currently a widely used animal model for identifying the neural circuits and molecular pathways involved in adult amblyopia and visual plasticity (Hofer et al., 2006; Kang et al., 2013; Frantz et al., 2015). However, to date, very few studies in VPL have been performed in mice. Frenkel et al. (2006) previously reported that repeated presentations of specific grating stimuli resulted in a stimulus-selective response potentiation (SRP) in primary visual cortex (V1) of awake mice, similar to the VPL-induced increase in fMRI response in the human V1 (Furmanski et al., 2004). They found that SRP required NMDA receptor activation and AMPA receptor trafficking. Although SRP is generally not considered a standard form of VPL, owing to the lack of a specific visual task (Karmarkar and Dan, 2006; Bonaccorsi et al., 2014), these findings imply that the mouse will likely become an important and tractable model for revealing the mechanisms underlying VPL.

In this study, we thoroughly evaluated the effect of VPL in C57BL/6J mice using a two-alternative forced-choice visual water task. The mice were subjected to CS and VA assessments by discriminating between two orthogonal gratings (pattern discrimination), or detecting the presence of a single grating (visual detection). Then, the mice underwent repeated training at near the individual threshold of contrast or spatial frequency (SF) for 35 consecutive days. Following training, the mice exhibited significant improvements in CS and VA. We further examined the specificity and generalization of learning to the eye, stimulus orientation and task, as well as the effect of VPL on the recovery of VA in adult amblyopic mice and old mice. Using the mouse model, we further evaluated the cut-off SFs and dendritic spine density in V1 neurons. Our findings suggest that the characteristics of VPL in mice are similar to those observed in other species and V1 may be involved in VPL.

## Materials and Methods

### Animals

Male C57BL/6J mice (Vital River Laboratory, Beijing, China) aged 19 days (*n* = 15), 8 weeks (*n* = 189), 4 months (*n* = 108) and 15 months (*n* = 5) were used in this study. All animals were housed in groups under standard laboratory conditions with a 12/12 light-dark cycle, 21°C ambient temperature and 35% relative humidity, and were given food and water *ad libitum*. Behavioral training was conducted during the light phase of the activity cycle. All animal procedures in this study were performed in accordance with the University of Science and Technology of China Guide for the Care and Use of Laboratory Animals and were approved by the Institutional Animal Care and Use Committee at the University of Science and Technology of China.

### Behavioral Apparatus and Visual Stimulation

A modified visual water task described in previous studies was used (Prusky et al., 2000b; Prusky and Douglas, 2004). Briefly, this task was a two-alternative, forced-choice visual discrimination task, in which mice learned to associate a sinusoidal grating with escape from water. The apparatus consisted of a trapezoidal-shaped (140 cm L × 100 cm W × 30 cm W) Plexiglas pool (55 cm H) and two 21-inch SONY G520 monitors (1024 × 768, 85 Hz) that were arranged side-by-side at the wide end of the pool. The pool contained 15 cm of water (22 ± 1°C) that was tinted white using powdered milk. The wide end of the pool was transparent, and the insides of the remaining walls of the pool were painted flat black to reduce light reflections within the pool. An opaque midline divider (48 cm L) was positioned along the centerline of the pool. The choice point, which was the closest point to the monitors that the mice could reach without entering one of the two arms of the pool, was set by the length of the divider. A positive stimulus (S+) was displayed on one monitor, while a negative stimulus (S-) was displayed on the other. A submerged escape platform (14 cm H) was placed below the monitor displaying the S+. Mice were released from a chute centered at the narrow end of the pool. Screen reflections on the surface of the water further made the platform invisible from the water level.

Visual stimuli were generated by computer software written in MATLAB (MathWorks, Natick, MA, USA) and displayed on gamma-corrected monitors. The mean luminance of stimuli was set at 43 cd/m^2^. The effective SF for the mouse was set by the length of the divider and was calculated by the computer software. The contrast was calculated using the screen luminance [(max-min)/(max + min)]. In this study, we used a pattern discrimination task consisting of a full-screen vertical sinusoidal grating (S+) vs. a full-screen horizontal sinusoidal grating (S-, V vs. H, see Results). Besides, to minimize edge effects, an approximately 4 cm half-Gaussian ramp was added to each side of the visual stimuli to blend the stimuli with the background (Gaussian). The visual stimuli consisted of circular sinusoidal gratings 23 cm in diameter. When the mice viewed the apparatus from the release chute of the pool at a distance of 100 cm, the grating extended over 13°, and the Gaussian-blurring ramp extended over another approximately 2°; at the choice line point at 48 cm, the apparent size of the grating and the blurred edge were approximately 26 and 4°, respectively. Moreover, in the VA task, we also used visual detection consisting of a full-screen vertical or horizontal sinusoidal grating (S+) vs. homogeneous gray background (S-, V vs. G or H vs. G). The homogeneous gray background was generated by setting the contrast level to zero.

### Behavioral Assessments of CS and VA

Behavioral assessments of CS and VA were adapted from previous studies (Prusky et al., 2000b; Prusky and Douglas, 2003, 2004). CS is equal to 1/contrast-threshold. In brief, the mice were first conditioned to associate swimming to the submerged platform with the S+ at a low SF (0.12 cycle per degree, cpd) and 100% contrast (training phase). If the mouse swam to the platform without entering the arm of the pool displaying the S-, the trial was recorded as correct; if the mouse swam to the arm with the monitor presenting the S-, the trial was considered an error. Once an incorrect choice was made, the mouse was immediately required to perform another trial, until a correct choice was made. To eliminate the possibility of side-biases in responses, the alternating pattern of the S+ and platform position was a pseudorandom pattern, in which no more than three consecutive trials were allowed on one side. Mice were usually tested in groups of five to eight in a session of 10 interleaved trials. Each session lasted approximately 1 h and two sessions were performed in a single day. In general, 60–70 trials were required for the mice to achieve near-perfect (90% or better) performance over three consecutive days. The swimming paths were recorded with a digital camera mounted above the pool and analyzed offline with Ethovision XT 8.5 (Noldus, Info Tech, Wageningen, Netherlands). Following the training phase, CS or VA was assessed.

For CS assessment, small decremental changes in the grating contrast were made within blocks of trials, until the accuracy fell below 70%. This procedure was initiated at 100% contrast, and decreases in contrast were made in steps of 10%. Typically, if the mouse made an error choice, a criterion test was initiated, in which additional trials were run at the same contrast until four consecutive correct choices were made in sequence, or seven correct choices were made in a block of 10 consecutive trials. A preliminary contrast threshold was established when a mouse failed to achieve 70% performance at a specific contrast value. To determine the validity of this estimate, the contrast of the grating was increased in steps, and the procedures were repeated several times until a stable pattern of performance was established (**Supplementary Figure S1A**). The accuracy at each contrast level was averaged, and a frequency-of-seeing curve was constructed in which the percentage of correct choices was plotted against the contrast. The point at which the curve intersected with 70% accuracy was recorded as the contrast threshold, which was converted to the CS by taking the reciprocal. The CS functions (CSFs) of the naïve mice were generated by measuring the CSs at SFs of 0.06, 0.12, 0.21, 0.33, 0.42, and 0.46 cpd. To reduce the potential impact of measuring the contrast threshold at one SF on the performance of the neighboring SF, each mouse was only tested at a single SF. Similarly, the CSFs of the trained mice were obtained by measuring the CSs at a wide range of SFs (0.06, 0.12, 0.21, 0.33, 0.42, 0.57, and 0.72 cpd).

Visual acuity was assessed with the same procedure and criterion testing used in the CS assessment, with the exception that the test began with a low SF (0.12 cpd) grating. Briefly, the SF of grating was increased incrementally within blocks of trials in a step of 0.03 cpd when four consecutive correct choices were made in sequence, or when seven correct choices were made in a block of 10 consecutive trials. A preliminary SF threshold was attained for the mice until they failed to achieve 70% performance. As with contrast threshold assessment, the SF of the grating was decreased in steps between blocks of trials until the accuracy was equal to or greater than 70%. The process was repeated several times until a stable pattern of performance was established (**Supplementary Figure S1B**). The performance at each SF was averaged and used to construct a frequency-of-seeing curve in which the correct percentage was plotted against the SF. The VA was recorded as the SF corresponding to 70% accuracy on the curve.

### Perceptual Training Procedures

The training procedures for VPL consisted of three consecutive phases: pre-training assessment, perceptual training, and post-training reassessment. The testing and training procedures were identical for the pre-training, perceptual training, and post-training sessions, as described above. Following an assessment of the contrast or SF threshold, mice received near-threshold training in CS or VA tasks for 35 consecutive days. Two sessions were performed on a single day, except when otherwise noted.

Briefly, for VPL of CS, the grating contrast was varied near the individual contrast threshold (NCT training) at a SF of 0.33 cpd within blocks of trials in the V vs. H task. When mouse failed to achieve 70% accuracy at the contrast threshold, the contrast was increased in steps until greater than 70% accuracy was achieved, and vice versa. For VPL of VA, the grating SF was varied near the individual SF threshold (NSFT training) at 100% contrast within blocks of trials in pattern discrimination or visual detection tasks. Once a mouse failed to achieve 70% accuracy at the SF threshold, the SF was decreased in steps until greater than 70% accuracy was achieved, and vice versa. The post-training CS and VA were averaged over the final four training days. For the control groups, the overall swim time and training days were matched to those of the NCT or NSFT group, except that the grating was at 100% contrast or 0.12 cpd.

### Sucrose Preference Test

To assess the effect of long-term training on sucrose preference, mice were given a free choice for 24 h of two drinking bottles: one with 1% sucrose solution, and another with tap water, as previously described (Ribeiro-Carvalho et al., 2011; Martinez-Hernandez et al., 2012). To enhance the drinking behavior, the mice were deprived of water for 24 h prior to the start of the sucrose preference test. The position of the bottles in the cage was interchanged after 12 h, to eliminate the potential effects of side-preference on drinking behavior. Special precautions were taken to minimize liquid spills and errors in measurement during the tests. The consumption of sucrose solution and water was recorded simultaneously in all groups of mice by weighing the bottles. The percentage preference for sucrose was determined using the following formula: sucrose preference = (sucrose consumption)/(water consumption + sucrose consumption) × 100%.

### Intrinsic Signal Optical Imaging and Data Analysis

Intrinsic signal optical imaging was performed in the mouse visual cortex as described in our recent study (Zhang et al., 2015). Mice were initially anesthetized with ketamine (0.1 mg/g) and xylazine (0.01 mg/g) mixture (i.p.), and then were maintained by isoflurane (0.5–1%). Briefly, the cortex was illuminated with a cool light at 550 nm to obtain the brain vasculature map and at 720 nm to collect evoked signals. The images were captured using a Dalsa Pantera 1M60 CCD camera with a resolution of approximately 17 μm/pixel. Visual stimuli were created by MATLAB (MathWorks, Natick, MA, USA) using the Psychophysics Toolbox and presented on a gamma-corrected LCD monitor with a mean luminance of approximately 32 cd/m^2^. The monitor was placed 24 cm in front of the animal. To assess the cut-off SF in V1, the sinusoidal gratings were displayed in binocular visual field (-5 to +15° horizontal by -15 to +45° vertical). For mapping retinotopy, a white bar was moved in two opposite directions along each cardinal axis for 10 min periodically at 0.1 Hz (**Supplementary Figure S4A**), as previously described (Cang et al., 2005). We computed the response magnitudes by extracting the optical signal at the stimulus frequency and analyzed the phase scatter of the retinotopic maps to assess the map quality. Fourier analysis was used to extract two matrices pixel by pixel: one matrix for response strength at a given stimulus frequency and the other matrix representing stimulus eccentricity. For single-condition maps, drifting gratings were displayed for 4 s. A gray screen was shown during the pre-stimulus baseline period (1 s) and between trials (7 s). Frames that were obtained after the stimuli and displayed on for 2–6 s were averaged, and then a blank frame (the average response for a 1 s interval prior to the stimuli onset) was subtracted and divided to generate a single-condition map of reflectance change (ΔR/R). To avoid signal distortion, all of the images were smoothed (kernel of 3 pixels or 51 μm in radius) by a circular averaging filter. Finally, all of the filtered ΔR/R maps under the same stimuli conditions across 32 trials were averaged to obtain a single-condition map. The cut-off SF was evaluated by the zero-crossing of a linear fit (see the example in **Figure 5B**).

### Golgi-Cox Stain Analysis

The mice were deeply anesthetized with pentobarbital (60 mg/kg, i.p.) and intracardially perfused with 0.9% saline after the end of the training period. The brains were removed from the calvaria and stored in a Golgi-Cox solution in the dark at room temperature for 14 days according to published procedures (Gibb and Kolb, 1998). Then, the brains were transferred to a 30% sucrose solution for 4 days and were coronally sectioned (200 μm) at the V1 level using a vibratome. The sections were mounted onto microscope slides that were coated with polylysine. After 2 days, the brain slices were stained with 10% ammonium hydroxide for 30 min followed by washing. Then, the slices were transferred to Kodak Film Fixer solution for another 30 min, rinsed with water, dehydrated, cleared, and cover-slipped according to standard procedures. The following criteria were used to select pyramidal neurons for analysis: (1) the neurons were well impregnated and not obscured by blood vessels or large clusters of neighboring dendrites, and (2) the apical and basilar dendrites of the neurons were largely intact and visible in the plane of the section. For each mouse, six to eight pyramidal neurons in layer 2/3 of V1 were traced using a camera lucida at a magnification of 200×. For the spine density analysis, the terminal secondary and tertiary dendrites were then traced and recorded with the camera lucida at 640×. The spine density was calculated for the chosen dendrite segments (at least 25 μm, measured by the Image-Pro Plus 6.0) and presented as the number of spines per 10 μm. **Figure 5E** shows a representation of Golgi-stained dendritic spines. The statistical data were analyzed by individuals who were blinded to the experimental groups.

### Eyelid Suture

Monocular deprivation was performed through the eyelid suturing of mice under 0.5–2% isoflurane anesthesia. The eyelids were trimmed and an antibacterial ophthalmic agent was applied to the eye. Two mattress stitches were placed using 7–0 silk. Following surgery, the mice were administered an antibiotic chlortetracycline eye ointment on the surface of the wound and were then allowed to recover on a warm blanket. The mice underwent long-term monocular deprivation from P19 to P70 and a reverse suture, consisting of reopening the deprived eye and closing the fellow eye from P70 to P115. Great care was taken to prevent opacities of the reopened eye by applying chloramphenicol eye drops onto the cornea. The mice were checked daily after surgery, and those showing spontaneous lid reopening or eye anomalies were excluded from the following study.

### Statistical Analysis

All data were expressed as means ± SEM. Statistical analyses were conducted using IBM SPSS Statistics 21 software (SPSS Inc., Chicago, IL, USA). One-way ANOVA, two-way mixed-design ANOVA and repeated-measures ANOVA were applied to the data as appropriate. When the initial test yielded a significant interaction, Tukey’s *post hoc* test was used. The pre-training and post-training values were compared within each group using a paired Student’s *t*-test. Differences between two groups were assessed with a two-tailed *t*-test. Statistical significance was set as ^∗^*p* < 0.05, ^∗∗^*p* < 0.01, ^∗∗∗^*p* < 0.001. The percent improvement was calculated as [(post-training value–pre-training value)/(pre-training value)] × 100%. The retention coefficient of VA was defined as (VA_retest_–VA_pre_)/(VA_post_–VA_pre_) × 100%. The transfer index (TI) was defined as (VA_post_–VA_naive_)_untrained_/(VA_post_–VA_pre_)_trained_. Note that TI = 1 indicates complete transfer, and TI = 0 indicates no transfer.

## Results

### VPL Improves CS in Mice

**FIGURE 1:**
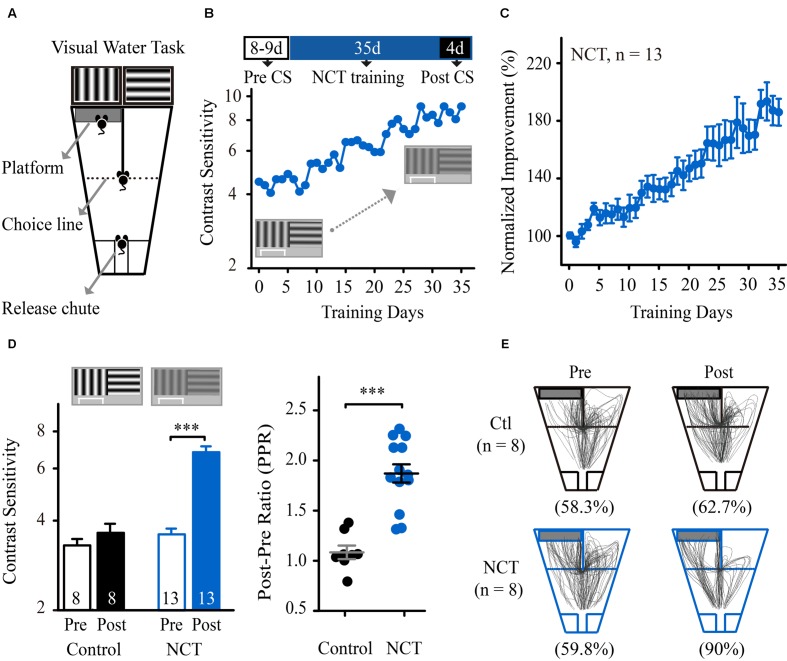
**Training at near-contrast threshold in a pattern discrimination task resulted in a significant improvement in CS.** **(A)** Diagram of the modified version of the visual water task. Following release from the chute, the mice learned to swim toward the monitor displaying a vertical grating to find the submerged platform and escape from the water. Modified from Prusky et al. (2000b). (**B**; Top) Schematic of the experimental procedure. The mice were subjected to CS assessment for 8–9 days and then prolonged training at the NCT for 35 days. The post-training CS was averaged during the final four training days. (Bottom) CS at the trained SF vs. training days of one representative mouse in the NCT group. **(C)** The average learning curve for mice in the NCT group across training days. The CS was normalized to the individual pre-training CS. (**D**: Left) The average CS that was obtained from mice in the control group trained at 100% contrast and from the NCT group before (Pre) and after (Post) training. The numbers in the columns indicate the number of animals included in each group. (Right) The post/pre ratios (PPR) of mice in the control and NCT groups. Data from individual mice are plotted; long line represents the group average, and short lines represent ±SEMs. **(E)** Swim paths of mice in the control and NCT groups tested at a given grating contrast (20%) before and after training. For data analysis, all of the paths with the platform positioned on the right side of the maze were horizontally flipped around a vertical axis (mirror image) and combined with those on the left side of the maze. The numbers in parentheses indicate the average accuracy for each group before or after training. N, animal number. Data are expressed as means ± SEM. ^∗∗∗^*p* < 0.001.

We first determined whether perceptual learning improved the spatial sensitivity of mice for discriminating contrast-defined gratings. Twenty-one mice (aged 8 weeks) were subjected to a contrast threshold assessment in a pattern discrimination task (**Figure 1A**). The task consisted of training the mice to swim toward a vertical grating of 0.33 cpd (S+) vs. a horizontal grating of 0.33 cpd (S-, V vs. H task, see Materials and Methods). The average CS of these mice at the SF of 0.33 cpd was 3.75 ± 0.11, which is consistent with previous results (Prusky and Douglas, 2004). Then, the mice were randomly divided into two groups and subjected to repeated training at the SF of 0.33 cpd for 35 consecutive days. One group was trained at the NCT (NCT group), and the other group was trained at 100% contrast (control group). In **Figures 1B,C**, mice in the NCT group exhibited a gradual and significant increase in CS across training days. The average CS of these mice increased by 87% at the trained SF (from 3.75 ± 0.16 before to 7.05 ± 0.33 after training, **Figure 1D** left). Two-way mixed-design ANOVA with the group (control vs. NCT) as a between-subjects factor and test (Pre vs. Post) as a within-subjects factor revealed significant effects of the group [*F*_(1,19)_ = 44.885, *p* < 0.001], test [*F*_(1,19)_ = 75.479, *p* < 0.001], and their interaction [*F*_(1,19)_ = 52.568, *p* < 0.001]. These results indicated a greater benefit for CS in the NCT group than in the control group over the course of training. We quantified the magnitude of learning and expressed it as the ratio of the post-training value to the pre-training value (PPR). The mean PPR for mice in the NCT group (1.87 ± 0.10) was significantly higher than that in the control group (1.09 ± 0.07, *t*-test, *p* < 0.001, **Figure 1D** right). Moreover, following training, mice in the NCT group exhibited more correct swim paths while locating the escape platform and a higher average accuracy at 20% contrast than mice in the control group (*p* < 0.001, **Figure 1E**). Because the control mice were matched to the NCT mice in terms of overall swim time in the visual water maze, their lack of improvement in CS and VA indicates that the physical exercise component associated with a simple visual discrimination task does not contribute to the improvement in spatial vision.

We next determined whether prolonged training induced physiological changes in the animals. First, for mice in the NCT and control groups, body weight gains from the onset of training until the end of training were not significantly affected by the training, as the gains were comparable to those of naïve mice [two-way mixed-design ANOVA, group × test, *F*_(2,34)_ = 0.186, *p* = 0.831, **Supplementary Figure S2A**]. Secondly, no difference was observed in the swim speed between the two groups before and after training [two-way mixed-design ANOVA, *F*_(1,24)_ = 0.958, *p* = 0.337, **Supplementary Figure S2B**]. Finally, to exclude that stress from swimming may possibly contaminate our results, the mice were submitted to a sucrose preference test after the end of the training period. A lack of sucrose preference in rodents is considered a reliable index of anhedonia, a key symptom of depression. We found no statistically significant difference in sucrose preference among the naïve, control and NCT groups [one-way ANOVA, *F*_(2,26)_ = 0.09, *p* = 0.91, **Supplementary Figure S2C**). Moreover, no significant difference between the two groups was found for the escape latency to reach the platform during the post-training trials (NCT group: 4.2 ± 0.07 s, control group: 3.5 ± 0.09 s; *t*-test, *p* = 0.15). Obviously, the latencies presented here are far less than those that have been used in forced swimming tests (usually 3–15 min), which are often used to produce a rodent model of despair or depression. Collectively, these data indicate that training-induced improvement in performance is not due to physiological changes in mice.

In summary, these results show that repeated training at near the contrast threshold for pattern discrimination can result in a significant improvement in CS at the trained SF in mice, similar to the features that have been observed in humans (Polat et al., 2004; Zhou et al., 2006) and larger animal models (Hua et al., 2010; Chen et al., 2013).

### VPL Improves VA in Mice

**FIGURE 2:**
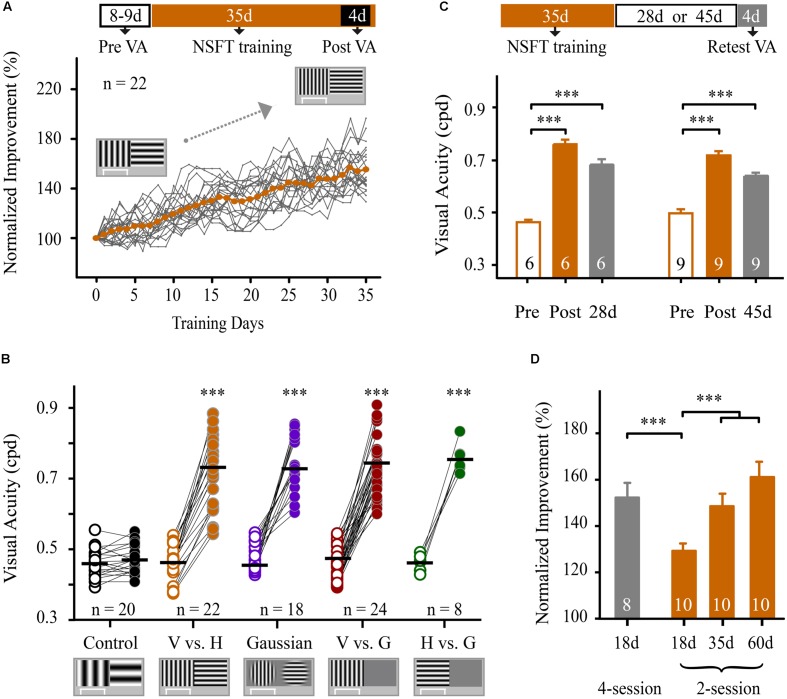
**Training at the near-spatial frequency threshold in pattern discrimination and visual detection tasks resulted in a significant and long-lasting improvement in VA.** (**A**; Top) Schematic of the experimental procedure. The mice were subjected to VA assessment for 8–9 days, followed by training at the NSFT for 35 days. The post-training VA was averaged during the final four training days. (Bottom) Normalized improvements in VA of all mice in the NSFT group for pattern discrimination (vertical grating vs. horizontal grating, V vs. H) are plotted as a function of training days. The VA was normalized to the individual pre-training VA. The gray lines represent individual mice, whereas the orange dotted line shows the group average. **(B)** The average pre- and post-training VA in the control mice that were trained at SF 0.12 cpd and in the four groups of mice that received 35 days of NSFT training in V vs. H, Gaussian-ramp masked V vs. H (Gaussian), vertical grating detection (V vs. G) and horizontal grating detection (H vs. G) tasks. The horizontal bar indicates the mean value. Linked symbols represent the pre- (open) and post-training VAs (solid) in the same eye of a mouse. The significance was compared for each group. N, animal number. **(C)** The average pre- and post-training VA as well as the retested VA at 28 or 45 days after training obtained from mice in two NSFT groups. **(D)** Normalized improvement in the VA of mice that received NSFT training in the 2-session (2 sessions per day) and 4-session (4 sessions per day) groups during different training periods. The numbers in the columns indicate the number of animals in each group. Data are expressed as means ± SEM. ^∗∗∗^*p* < 0.001.

We next determined whether perceptual training also improved spatial discriminability for identifying SF-defined gratings in mice. Before training, forty-two mice underwent VA measurement in the V vs. H task as described above. The average VA of these mice was 0.47 ± 0.01 cpd, which is consistent with previous studies (Prusky et al., 2000b; Prusky and Douglas, 2004). These animals were then randomly assigned to two groups for 35 days of training: one group underwent perceptual training at the NSFT (NSFT group), and the other group underwent training at a low SF (0.12 cpd, control group). Two-way mixed-design ANOVA (group × test) revealed the main effects of the group [*F*_(1,40)_ = 51.076, *p* < 0.001] and test [*F*_(1,40)_ = 182.667, *p* < 0.001], as well as an interaction of group × test [*F*_(1,40)_ = 156.392, *p* < 0.001]. These results indicate greater improvement of VA in the NSFT group than in the control group over the training period. **Figure 2A** shows that the mice in the NSFT group exhibited a gradual improvement in VA across training days. Following training, the average VA increased by 53.5% (paired *t*-test, *p* < 0.001, V vs. H group, **Figure 2B**). In contrast, no significant increase in VA was observed in the control group (paired *t*-test, *p* = 0.63). The absolute values are listed in **Table 1**.

**Table 1:** Behavioral improvements in visual acuity in different training groups.

Training group	*n*	Pre-VA	Post-VA	Post-VA/Pre-VA (PPR)
V vs. H	22	0.46 ± 0.01	0.72 ± 0.02^∗∗∗^	1.54 ± 0.04
Gaussian	18	0.48 ± 0.01	0.75 ± 0.02^∗∗∗^	1.56 ± 0.06
V vs. G	24	0.46 ± 0.01	0.74 ± 0.01^∗∗∗^	1.62 ± 0.04
H vs. G	8	0.47 ± 0.02	0.75 ± 0.04^∗∗∗^	1.63 ± 0.06
Control	20	0.46 ± 0.01	0.47 ± 0.01	1.02 ± 0.01


*Data are presented from four groups of mice that received 35 days of NSFT training in the V vs. H, Gaussian, V vs. G and H vs. G tasks, and from the control group of mice that were trained at 0.12 cpd in the V vs. H task. N, animal number; Pre-VA, pre-training visual acuity; Post-VA, post-training visual acuity. Data are presented as means ± SEM. ^∗∗∗^*p* < 0.001.*

We then determined whether NSFT training in visual tasks with other visual stimuli could also result in VA improvement in three additional groups of mice. Using a training procedure identical to that used in the V vs. H group, one group was subjected to training in a Gaussian-ramp masked V vs. H task (Gaussian group), and two other groups were subjected to training in vertical and horizontal grating detection tasks (V vs G group and H vs G group, respectively; see Materials and methods). All of the mice in the three groups showed significant improvements in VA after training with values of 55.7% in the Gaussian group, 62.4% in the V vs. G group and 63.2% in the H vs. G group (**Figure 2B** and **Supplementary Figure S3**, details in **Table 1**). For the four groups, no significant differences in VAs were induced by NSFT training [two-way mixed-design ANOVA, *F*_(3,78)_ = 1.289, *p* = 0.284], indicating that the different visual stimuli themselves had no major effect on VA improvements. These results indicate that prolonged training at near the SF threshold in both pattern discrimination and visual detection tasks leads to a significant improvement in VA in mice.

To evaluate the impact of the daily training intensity on the magnitude of the improvement of VA, we trained two additional groups of mice in the V vs. H task. One group received training in two daily sessions (2-session group), and the other group received training in four daily sessions (4-session group). After 18 days of training, the average VA improvement in the 4-session group (56 ± 7%) was significantly higher than that in the 2-session group (31 ± 4%, *p* = 0.004, **Figure 2D**). Two-way mixed-design ANOVA revealed the main effects of the group [*F*_(1,16)_ = 175.491, *p* < 0.001], test [*F*_(1,16)_ = 142.565, *p* < 0.001], and the group × test interaction [*F*_(1,16)_ = 17.098, *p* = 0.001]. These data indicate that an increase in daily training sessions facilitates the improvement in VA. Moreover, mice in the 2-session group were continually trained for 60 days. One-way repeated-measures ANOVA showed a significant main effect of the training days [*F*_(2,18)_ = 18.794, *p* < 0.001], indicating an improvement in VA over the training period. Tukey’s *post hoc* test revealed a significant improvement in VA at 18 days vs. 35 days (*p* = 0.004), but not at 35 days vs. 60 days (*p* = 0.165, **Figure 2D**). Thus, we used 35 days of training in this study.

To confirm that this VA learning was not a temporary adaptation effect, we evaluated the retention of VA improvement at 28 and 45-days follow-up examinations after the completion of perceptual training in two groups of NSFT mice (**Figure 2C**). For both groups, one-way repeated-measures ANOVA showed a significant main effect of the tested days [28-days group, *F*_(2,10)_ = 104.324, *p* < 0.001; 45-days group, *F*_(2,16)_ = 50.181, *p* < 0.001]. The retested VAs of mice for each group were significantly greater than their pre-training VA (Tukey’s *post hoc* test: 28-days group, *p* < 0.001; 45-days group, *p* < 0.001), but were much lower than their post-training VAs (Tukey’s *post hoc* test: 28-days group, *p* = 0.012; 45-days group, *p* = 0.001). We defined a retention coefficient to determine the retention of the learning effect. The retention coefficients were 74 and 63% for the 28 and 45-days retested groups, respectively.

Therefore, training mice near the SF threshold for pattern discrimination and visual detection is sufficient to produce significant and long-lasting improvements in VA.

### CS Learning Transfers to VA and Vice Versa

**FIGURE 3:**
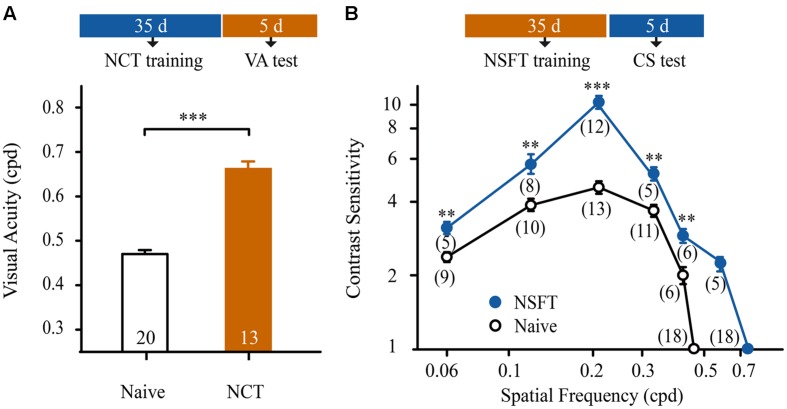
**Contrast sensitivity learning was transferred to improvements in VA and vice versa.** **(A)** Effect of NCT training on VA. Open and solid columns indicate the average VA of naïve and NCT mice. The numbers in the columns indicate the number of animals. **(B)** Effect of NSFT training on CS function. The averaged CS functions of the naïve mice are indicated by open circles and a black line; the averaged CS functions of the NSFT trained mice are represented by solid circles and a blue line. The numbers in parentheses indicate the number of analyzed mice. Data are expressed as means ± SEM. ^∗∗∗^*p* < 0.001, ^∗∗^*p* < 0.01.

We determined whether the improvement in performance could be transferred from a CS task to a VA task and vice versa. Following 35 days of training, the mice in the NCT group were subjected to VA assessment in the trained V vs. H task. These mice exhibited a 55% improvement in VA compared to age-matched naïve mice (*t*-test, *p* < 0.001, **Figure 3A**).

An additional 59 mice, following 35 days of NSFT training, received CS assessments at a wide range of SFs (0.06, 0.12, 0.21, 0.33, 0.42, 0.57, and 0.72 cpd) in the trained V vs. H task. For comparison, 67 naïve mice underwent CS assessments at 0.06, 0.12, 0.21, 0.33, 0.42, and 0.46 cpd. As expected, the mice in the NSFT group exhibited a general benefit in CS over a wide range of SFs compared to the naïve mice (**Figure 3B**). Two-way mixed-design ANOVA with group (NSFT vs. naïve) × SF (0.06–0.42 cpd) revealed the main effects of group [*F*_(1,75)_ = 74.659, *p* < 0.001], SF [*F*_(4,75)_ = 63.362, *p* < 0.001] and the group × SF interaction [*F*_(4,75)_ = 18.499, *p* < 0.001]. Tukey’s *post hoc* test indicated no significant difference in CS at 0.06 cpd vs. 0.42 cpd (*p* = 0.89) or 0.12 cpd vs. 0.33 cpd (*p* = 0.94). In contrast, significant differences in CS were observed at 0.21 cpd vs. all of the other SFs tested (all, *p* < 0.001). Taken together, the maximal improvement in the CS of NSFT mice was observed at the SF of 0.21 cpd (from 4.59 ± 0.27 to 10.82 ± 0.08, *p* < 0.001). Thus, prolonged training at the NCT or NSFT is highly effective for improving spatial vision in normal mice.

### Specificity and Generalization of VPL to Eye, Orientation and Task

**FIGURE 4:**
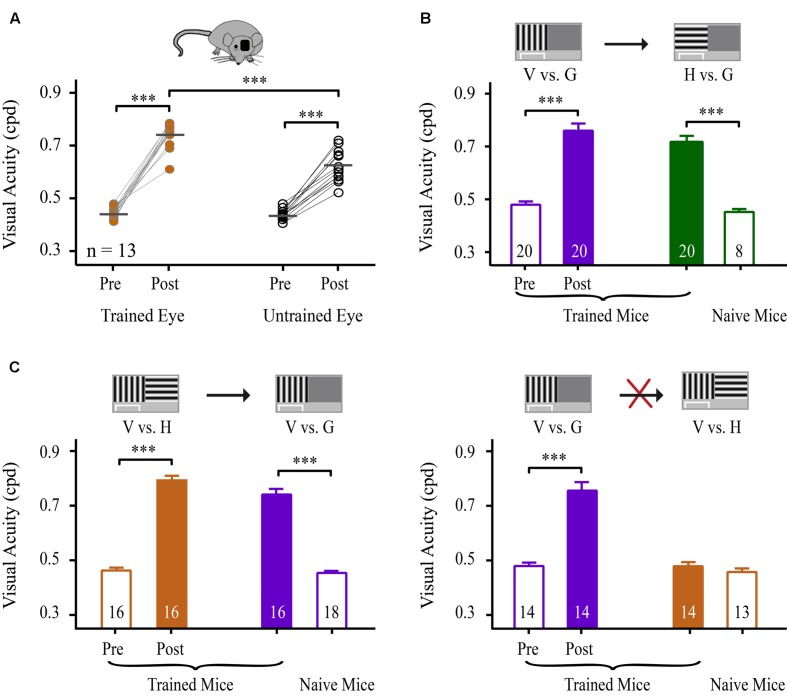
**Specificity and generalization of learning to the eye, orientation and task.** **(A)** Effects of learning on VA for the trained and untrained eyes. Scatter plots represent the pre- and post-training VA in the trained (left panels) and untrained (right panels) eyes of mice that received monocular NSFT training in the V vs. H task for 35 days. The horizontal bar represents the mean value. Linked symbols represent the pre- and post-training VAs in the same eye of a mouse. N, number of animals. **(B)** Effects of learning on the VA for the trained vs. untrained orientations. The mice received 35 days of NSFT training in the V vs. G task (purple column), followed by VA assessment in the untrained H vs. G task (solid green column). The open green column indicates the VA of naïve mice in the H vs. G task. **(C)** Effects of learning on the VA for the trained and untrained tasks. Learning in the V vs. H task (orange column) was significantly transferred to the untrained V vs. G task (purple column), but not vice versa. Mice underwent 35 days of NSFT training. (Left) The pre- and post-training VA in the trained V vs. H task and VA in the untrained V vs. G task. The open purple column indicates the VA in the V vs. G task of the naïve mice. (Right) The pre- and post-training VA in the trained V vs. G task and VA in the untrained V vs. H task. The open orange column represents the VA of the naïve mice in the V vs. H task. The numbers in the columns indicate the number of analyzed mice. Data are expressed as means ± SEM. ^∗∗∗^*p* < 0.001.

To determine whether learning could transfer to the untrained eye, we compared the magnitudes of the improvement in VA between the trained and untrained eyes of mice. Using an identical paradigm for binocular training, 13 mice underwent monocular NSFT training in the V vs. G task for 35 days. The untrained eyes of these mice were covered with a piece of black electrical tape while the animals performed the task. Prior to and following perceptual training, the mice were subjected to VA assessment of each eye. The average improvement in VA for these mice was 67% in the trained eye and 42% in the untrained eye (**Figure 4A**, **Table 2**). Two-way repeated-measures ANOVA revealed significant effects of test [*F*_(1,24)_ = 13.955, *p* = 0.001], eye [*F*_(1,24)_ = 304.680, *p* < 0.001] and the test × eye interaction [*F*_(1,24)_ = 14.237, *p* = 0.001], indicating that the trained eyes of mice exhibited significantly greater improvement in VA than their untrained eyes. Moreover, compared to binocular-trained mice, a main effect of the group × test interaction was observed [two-way mixed-design ANOVA, *F*_(2,59)_ = 7.206, *p* = 0.002]. Tukey’s *post hoc* test indicated no significant difference in VA improvement between the binocular-trained mice and the trained eye of monocular-trained mice (*p* = 0.98), but a significant difference was found between the binocular-trained mice and the untrained eye of monocular-untrained mice (*p* < 0.001). Thus, these results show that monocular training greatly improves the VA in the trained eye, with a certain degree of specificity for this eye and significantly partial transfer to the untrained eye.

**Table 2:** Specificity and generalization of learning to eye, orientation and task.

Trained eye/task	*n*	Pre-VA	Post-VA	Untrained eye/task	Pre-VA (naïve)	Post-VA
Trained eye (I)	13	0.44 ± 0.01	0.74 ± 0.02^∗∗∗^	Untrained eye	0.43± 0.02	0.62 ± 0.02^∗∗∗^
V vs. G (II)	20	0.45 ± 0.01	0.73 ± 0.02^∗∗∗^	H vs. G	0.46 ± 0.02 (*n* = 8)	0.73 ± 0.02^∗∗∗^
V vs. H (III)	16	0.47 ± 0.01	0.78 ± 0.02^∗∗∗^	V vs. G	0.46 ± 0.01 (*n* = 18)	0.74 ± 0.02^∗∗∗^
V vs. G (IV)	14	0.44 ± 0.01	0.74 ± 0.02^∗∗∗^	V vs. H	0.46 ± 0.01 (*n* = 13)	0.48 ± 0.01


*Visual acuity learning showed significant transfer across eye (I) and stimulus orientation (II). In addition, learning in the V vs. H task largely transferred to the untrained V vs. G task (III), but not vice versa (IV). N, animal number. In II, III and IV, the control VA in the untrained tasks was obtained from age-matched naïve mice. The numbers in parentheses indicate the number of animals that were used in the naïve group. Data are presented as means ± SEM. ^∗∗∗^*p* < 0.001.*

Next, we examined the specificity of learning for stimulus orientation. A TI was used to compare the transfer of the improvement in VA among different training conditions. Twenty mice completed 35 days of NSFT training in the V vs. G task. Then, the orientation of the grating was rotated by 90°, and new trials were applied to measure the VA in the untrained H vs. G task. A marked improvement in the VA in the H vs. G task was observed in these mice compared to naïve mice (*t*-test, *p* < 0.001), suggesting an almost complete transfer of learning to an orthogonal orientation (*TI* = 0.96, **Figure 4B**, **Table 2**).

We were also interested in determining whether learning in a pattern discrimination task could transfer to a visual detection task and vice versa. To test this, two groups of mice were subjected to 35 days of NSFT training in V vs. H and V vs. G tasks. Interestingly, following training in the V vs. H task, the mice showed a perfect transfer of the improvement in VA to the untrained V vs. G task (*TI* = 0.90, *p* < 0.001, **Figure 4C** left, **Table 2**). However, learning in the V vs. G task failed to generalize to the untrained V vs. H task (*TI* = 0.07, *p* < 0.001, **Figure 4C** right, **Table 2**). This asymmetry of transfer indicates that different neural mechanisms may underlie VPL in pattern discrimination and visual detection.

### VPL Enhances the Cut-off SFs and Dendrite Spine Density in V1

**FIGURE 5:**
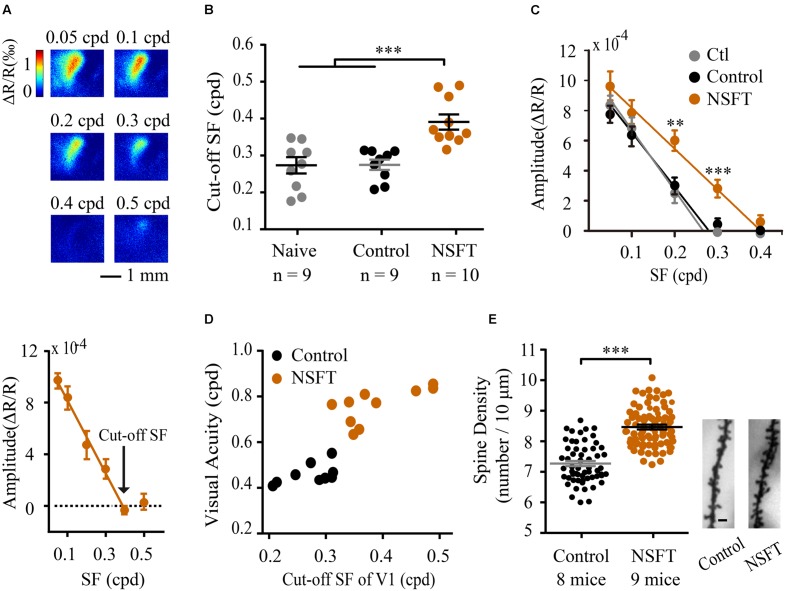
**Perceptual training enhanced the cut-off SFs and dendritic spine density in V1.** **(A)** Example V1 responses to the grating patches with a range of SFs from a NSFT mouse (top). Mean reductions in light reflectance in a selected region of the V1 binocular cortex (bottom). The graph depicts the means ± SD. **(B)** Cut-off SFs in V1 of individual mice in the naïve, control and NSFT groups as indicated by the different data points. Error bars, means ± SEM. **(C)** Average intrinsic signal responses of the mice in the three groups. *t*-test, ^∗∗^*p* < 0.01, ^∗∗∗^*P* < 0.001. **(D)** Scatter plots of behavioral post-training VA (x-axis) vs. the cut-off SFs in the mouse V1 (y-axis). Black and red circles represent the individual values for mice in the control and NSFT groups, respectively. **(E)** Comparison of the dendritic spine density in V1 layer 2/3 neurons of the NSFT and control mice. Each circle represents the spine density of each V1 neuron. A total of 58 neurons from 8 mice and 70 neurons from 9 mice were analyzed in the control group and NSFT group, respectively. Error bars, means ± SEM. The right panel shows the representative spines of the control and NSFT mice. Scale bar, 2 μm. ^∗∗∗^*p* < 0.001.

To measure the contribution of V1 to the training-induced improvement in VA, we evaluated the cut-off SFs of V1 in mice following perceptual training, as measured by *in vivo* intrinsic signal optical imaging. Examples of cortical retinotopic maps of a NSFT and a naïve mouse are shown in **Supplementary Figure S4**, in which the map scatter and response amplitude are plotted. No significant differences were found in the cortical retinotopy and response amplitude between the two groups, indicating that VPL has little impact on the basic retinotopic organization of V1. Then, we recorded the response magnitude as evoked by grating patches with different SFs and calculated the cut-off SFs by linear fitting for the naïve, control and NSFT mice (**Figures 5A,C**). When comparing the cut-off SFs of all groups, a significant effect of training was observed [one-way ANOVA, *F*_(2,25)_ = 12.625, *p* < 0.001]. The average cut-off SF of the NSFT mice (0.39 ± 0.07 cpd) was significantly higher than those of the control (0.27 ± 0.04 cpd) and naïve mice (0.27 ± 0.06 cpd; Tukey *post hoc* test, both *p* = 0.001; **Figure 5B**). The threshold value measured by optical imaging was consistent with that of our previous study (Zhang et al., 2015), which was lower than that determined by behavioral measurements. Therefore, we plotted the behavioral VA and its cut-off SF in V1 for each mouse, which showed that the behavioral VAs were correlated with the cut-off SFs of V1 in all of the control and NSFT mice (**Figure 5D**). Thus, we conclude that the improved VA may be attributed, to a large degree, to the increased spatial discrimination ability of V1.

Changes in function are usually accompanied by persistent structural changes, which have not been addressed in VPL. Layer 2/3 pyramidal neurons in V1 receive visual input from layer 4 neurons and are modulated by top-down input from higher brain regions (Ferster and Miller, 2000). We therefore studied the effect of VPL on dendritic spine density along the secondary and tertiary branches of layer 2/3 pyramidal neurons in V1. The average spine density (per 10 μm of dendrite) in the NSFT mice (8.40 ± 0.15) was 13% higher than that in the control mice (7.42 ± 0.14, *t*-test, *p* < 0.001, **Figure 5E**). Taken together, our results indicate that structural changes in V1 might participate in mouse VPL.

### VPL Completely Restores VA in Visually Impaired Mice

**FIGURE 6:**
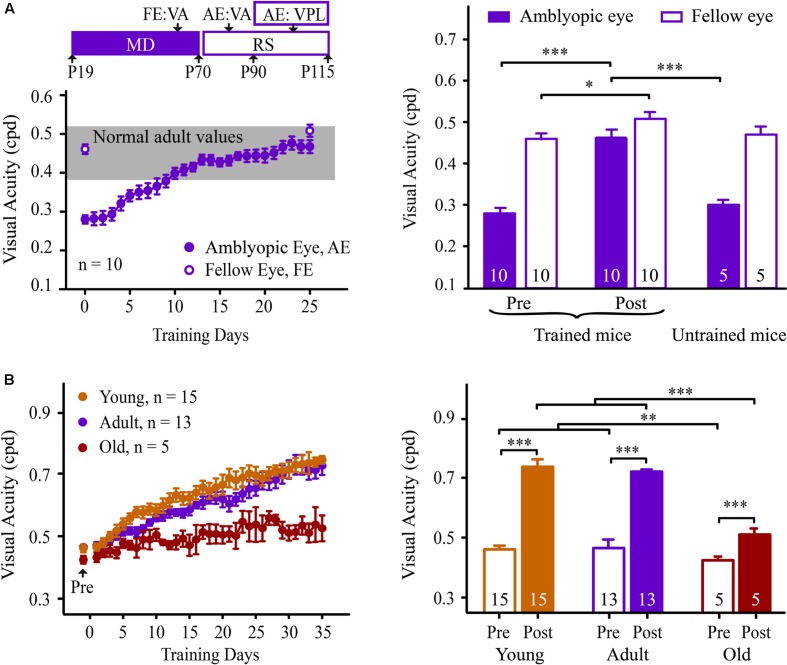
**Learning performance in adult amblyopic **(A)** and old mice **(B)**.** (**A**: Left) Experimental timeline for the measurement of the effect of the behavioral paradigm in the adult amblyopic mice (top). Mice received monocular deprivation (MD) from P19 to P70, followed by reverse suture (RS) from P70 to P115. The mice were subjected to VA assessments in the fellow eye (FE, P60-70) and the amblyopic eye (AE, P80-90). Then, the mice underwent NSFT training of the amblyopic eye. (Bottom) The average learning curve for the amblyopic eye of the adult amblyopic mice. Solid circles represent the VA in the trained amblyopic eye. Open circles represent the pre- and post-training VAs in the untrained fellow eye. The gray box denotes the range of VA in adult normal mice. (Right) The pre- (left columns) and post- (middle columns) training VA in the amblyopic and fellow eyes of the trained adult amblyopic mice, and the VA (right columns) in each eye of age-matched untrained adult mice with amblyopia. (**B**: Left) The average learning curves for mice in the young (orange), adult (purple) and old (red) groups. All of the mice received 35 days of NSFT training. (Right) The average VA of mice in the three groups before and after training. Data are expressed as means ± SEM. N, animal number. ^∗^*p* < 0.05, ^∗∗^*p* < 0.01, ^∗∗∗^*p* < 0.001.

To confirm the potential value of perceptual learning, we determined whether this type of learning could recover VA in adult mice with amblyopia. Fifteen mice were subjected to monocular deprivation from P19 to P70, followed by a reverse suture (**Figure 6A**). Before and after the reverse suture, mice underwent VA assessment for the fellow (non-deprived) and amblyopic (deprived) eye in the V vs. H task, respectively. Owing to long-term visual deprivation, mice showed a lower VA in the amblyopic eye (0.28 ± 0.01 cpd) than that in the fellow eye (0.46 ± 0.02 cpd, *p* < 0.001, **Figure 6A**), which is consistent with a previous report (Prusky and Douglas, 2003). When the mice received NSFT training of their amblyopic eye, the VA in their amblyopic eye showed a gradual and significant improvement across training days, reaching the normal range after training (0.47 ± 0.02 cpd, **Figure 6A**). In contrast, the age-matched amblyopic mice without training showed no significant improvement in the VA in their amblyopic eyes (0.30 ± 0.02 cpd, *p* = 0.13). Two-way mixed-design ANOVA showed significant effects of the group [*F*_(1,13)_ = 37.704, *p* < 0.001], test [*F*_(1,13)_ = 21.526, *p* < 0.001] and their interaction [*F*_(1,13)_ = 17.916, *p* = 0.001]. These results indicated that the improved VA was due to perceptual training *per se* rather than visual experience after reverse suture. Furthermore, the fellow eye of the trained mice also exhibited a small amount of improvement in VA (*p* = 0.02), indicating that the recovery of VA in the amblyopic eye did not occur at the expense of the spatial discriminability in the fellow eye. Therefore, perceptual learning is highly effective in the treatment of amblyopia in adult mice.

We further determined whether this VPL persisted in old mice. Fifteen adult (aged 4 months) and five old (aged 15 months) mice underwent VA assessments. A significant effect of age on VA was observed [one-way ANOVA, *F*_(2,32)_ = 5.627, *p* = 0.008]. The old mice exhibited significant decreases in VA compared to the young and adult mice (Tukey’s *post hoc* test, *p* = 0.011, **Figure 6B**). Following training, two-way mixed-design ANOVA revealed significant effects of the group [*F*_(2,32)_ = 36.835, *p* < 0.001], test [*F*_(1,30)_ = 249.083, *p* < 0.001] and the group × test interaction [*F*_(2,30)_ = 17.055, *p* < 0.001]. No significant difference was observed in VA improvement between the adult and young mice (Tukey’s *post hoc* test, *p* = 0.991, **Figure 6B**). Unlike the adult mice, the old mice showed a learning curve with a low slope across training days. However, the average VA of the old mice following training was not significantly different from those of the young and adult mice prior to training [one-way ANOVA, *F*_(2,32)_ = 1.272, *p* = 0.295]. These results suggest that VPL can be used as a possible intervention to counteract age-related declines in spatial vision.

## Discussion

In the present work, we have shown that VPL is well conserved in the mouse visual system. Following near-threshold training, the mice exhibited significant and long-lasting perceptual benefits in spatial vision, similar to those in humans and larger animal models. This VPL also promoted the recovery of normal VA in both adult amblyopic and old mice. The visual improvement that was observed in our study is not due to environmental enrichment, which is another strategy for visual improvement in adulthood (Cancedda et al., 2004; Sale et al., 2007). The magnitude of VA improvement in mice that were raised in an enriched environment was approximately 18% (Prusky et al., 2000a) compared to approximately one-third in our results. Using the mouse model, we further revealed a close relationship between VPL and cortical changes in V1.

This is the first study to thoroughly evaluate the properties of VPL in mice. Previous studies in rats (Hager and Dringenberg, 2010; Sale et al., 2011; Baroncelli et al., 2012; Kang et al., 2014) also investigate the mechanisms underlying VPL using different training tasks, such as SF discrimination task, while most of them leave the generalization and specificity of VPL out. We demonstrated that: (1) training of CS improved VA and vice versa; (2) improvements in VA partially transferred to the untrained eye; (3) learning transferred to an orthogonal orientation; (4) learning transferred from a pattern discrimination task to a visual detection task, but not vice versa. Interestingly, Sale et al. (2011) found that their VPL in SF discrimination task was specific for stimulus orientation, while the improvement of VA in adult amblyopic rats transferred to the orthogonal orientation (Baroncelli et al., 2012; Bonaccorsi et al., 2014). The discrepancy indicates that VPL in different tasks may have different underlying mechanisms and require different cortical regions involved (Lu et al., 2011; Kumano and Uka, 2013).

Many behavioral studies that have characterized VPL in humans and larger animals are highly relevant to our observations in the mouse model. First, human studies have demonstrated that repeated practice of CS and SF discrimination tasks such as grating orientation discrimination, letters identification and video games has produced significant improvements in CS and VA (Polat et al., 2004; Zhou et al., 2006; Astle et al., 2010; Chung et al., 2012; Hussain et al., 2014). Similarly, significant perceptual gains in CS and VA have been observed in mice following near-threshold training in different visual discrimination tasks. Second, the learning effect of VPL lasts for several months to 2 years in adult humans (Polat et al., 2004; Zhou et al., 2006; Polat, 2009). We found that the improvement in VA outlasted the end of the perceptual training by at least 28 days, corresponding to 2–3 years or more in the timescale of human life (Baroncelli et al., 2012; Bonaccorsi et al., 2014). Third, in most cases, VPL transfers completely or partially to the untrained eye in humans and monkeys (Levi et al., 1997; Chung et al., 2006; Zhou et al., 2006; Li et al., 2008). In cats, training in contrast orientation identification also leads to visual improvement in the untrained fellow eye (Hua et al., 2010). Consistent with these studies, this VPL in mice showed a partial interocular transfer. Finally, in agreement with evidence on human subjects (Levi, 2005; Chung et al., 2006; Bower and Andersen, 2012; DeLoss et al., 2015), a marked recovery of visual functions was evident in the adult amblyopic mice and old mice subjected to VPL. Therefore, our results in the mouse model complement many similar findings of VPL in humans and larger animal models.

The visual water task has long been used to assess the properties of spatial vision in rats and mice (Prusky et al., 2000b; Robinson et al., 2004; Wong and Brown, 2006; Lehmann et al., 2012). The CS and VA of mice obtained in our study are comparable to those obtained in previous studies (Prusky et al., 2000b; Cancedda et al., 2004; Prusky and Douglas, 2004; Corbetta et al., 2008; Wang et al., 2009; Stephany et al., 2014). However, it should be noted that the measured contrast threshold in mice varies with different behavioral paradigms (21%, Busse et al., 2011; ∼15%, Prusky and Douglas, 2004; 6%, Lehmann et al., 2012, Yeritsyan et al., 2012; 4%, Prusky et al., 2004; 2%, Histed et al., 2012; 1%, van Alphen et al., 2009). The differences might be due to many factors, such as the illumination of stimuli and the background, spatial summation, the motivational state and stress level of the animal, and the time and trial number when performing the measurement. Go/no-go tasks under mice head-fixation can yield hundreds of trials per day and can be readily combined with neurophysiological or calcium imaging recordings simultaneously. However, it takes 2 weeks for the mice to recover from head surgery and 3–4 weeks of training to learn the task, and it yields estimates of varying quality across sessions (Andermann et al., 2010; Histed et al., 2012; Glickfeld et al., 2013; Pinto et al., 2013). By comparison, the visual water task, in which mice are free-moving and require only 3 days of training to learn the task, is more robust, easier, and quicker. Because of the comparatively small investment of time and effort, the visual water task will have a broader benefit to the study of VPL during adolescence and development. Moreover, our study showed that hundreds of near-threshold trials were sufficient to improve visual functions in normal and amblyopic mice. Thus, one may consider the possibility: by measuring visual capabilities ever more precisely using more trials, experimenters may actually train the animals and improve their values. An appropriate control group may be served to avoid this problem. The alternative way is that one measures the value in few hours with electrophysiological or imaging methods, despite the fact that the relationship between the response of visual cortex and the behavior still requires further investigation.

The development of animal models represents a critical step for understanding the basic mechanisms in behavioral neuroscience. Whether the properties of V1 are changed after VPL is still in debate and presumably depends on the task (Schoups et al., 2001; Furmanski et al., 2004; Li et al., 2004; Carmel and Carrasco, 2008; Sasaki et al., 2010). The significant interocular transfer observed in mice indicates that the learning process more centrally takes place in the cortex, where the inputs from the two eyes converge. Since both anatomical and physiological data show that V1 is involved in processing detailed features of objects with the high CS and SF (Duncan and Boynton, 2003; Prusky and Douglas, 2004; Li et al., 2015), it is reasonable to speculate that VPL of VA requires neural changes in V1. Indeed, VPL continuously refines neuronal population codes in the monkey V1 (Yan et al., 2014). Hua et al. (2010) found that VPL improved the CS of V1 neurons in cats. Our results indicate that after the VA training, V1 populations responded to higher SFs. This result is consistent with a recent study (Poort et al., 2015) in which the authors observed a population-wide increase in neural selectivity in V1 layer 2/3 neurons when mice learned to discriminate two visual patterns. Moreover, VPL increased the dendritic spine density of layer 2/3 neurons in the mouse V1, which is consistent with previous results of mouse perceptual learning in the barrel (Kuhlman et al., 2014), auditory (Moczulska et al., 2013), and motor cortex (Xu et al., 2009). Taken together, these studies demonstrated the effects of VPL on the V1 neural responses. Meanwhile, the fact that learning transfers across orientations may reflect neural changes in a relatively high-level cortical areas where neurons are broadly tuned for orientation. Thus, these data may support the involvement of both the V1 and higher cortical areas in the mouse VPL. By selectively activating or inactivating distinct brain areas with optogenetic or pharmacologic techniques, future research on the mouse model will be able to reveal the causal relationships between neural circuits and VPL.

Visual perceptual learning has been regarded as a manifestation of cortical plasticity; however, the underlying molecular mechanism is largely unknown. For example, dopamine induces plastic changes in primary auditory cortex that could potentially support auditory perceptual learning (Bao et al., 2001). However, the role of dopamine in cortical plasticity during VPL has not been investigated. With the application of transgenic, optogenetic, and two-photon imaging tools, the mouse model will help to clarify how each neuron is affected by VPL and the molecular substrate of cortical plasticity during VPL. Furthermore, our studies have confirmed that VPL is effective for improving visual declines in mouse models of adult amblyopia. Numerous studies in mouse models have achieved significant progress in identifying the molecular substrates of adult amblyopia and providing intervention strategies for boosting brain plasticity in adulthood (Baroncelli et al., 2011; Bochner et al., 2014; Frantz et al., 2015). In combination with these findings, the use of our mouse model will help guide the development of a VPL-based rehabilitative approach.

Healthy aging is associated with a wide range of declines in vision, which has a profound effect on the health and well-being of older populations. Researchers have recently shown that low-vision older adults may benefit from VPL in many visual tasks, although the training benefits for older subjects are less than those for young adults (Bower et al., 2013; DeLoss et al., 2015). Our and previous studies showed reduced visual functions and visual cortex-dependent learning in old mice (Wong and Brown, 2007; Lehmann et al., 2012). Moreover, we found that VPL completely counteracted the age-related declines in the VA of old mice, despite the fact that aging largely affected VPL. The age-related declines in visual functions and learning might be due to the reduced cortical plasticity, reduced stability or increased noise in cortical processing (Schmolesky et al., 2000; Chang et al., 2014). However, it remains open for further investigation. Because mice have a short lifespan and hundreds of transgenic lines, another avenue of future research in the mouse model is to evaluate the mechanisms underlying age-related changes in VPL.

In summary, this study demonstrates that mice as a species exhibit reliable VPL, and this VPL is correlated with V1. We believe that the mouse model will serve as a valid and versatile tool to allow for a variety of mechanistically driven studies on VPL. These studies will eventually provide a more complete understanding of the mechanisms underlying VPL and guide the development of VPL as a potential approach for visual rehabilitation, thereby improving the quality of life of visually impaired individuals.

## Author Contributions

YW, YY, and YZ designed the research; YW, WW, XH, YL, SL, DM, XM, and HL conducted the behavioral experiments; YW, YY, YZ, and XA analyzed the behavioral data; YW performed the eyelid suture and Golgi-Cox staining experiments; XZ, WW, and JP performed intrinsic signal optical imaging and data analysis; YW and YY wrote the paper.

## Conflict of Interest Statement

The authors declare that the research was conducted in the absence of any commercial or financial relationships that could be construed as a potential conflict of interest.

## Supplementary Material

**FIGURE S1 | Behavioral measurements of CS **(A)** and VA **(B)**.** **(A)** Left figure, in the testing phase, the contrast of the grating (gray bars) was gradually decreased in steps. When the average performance (solid line) fell below 70% accuracy (dotted line), the contrast was increased, and the previous contrast levels were reassessed repeatedly. Right figure, a frequency-of-seeing curve for the mouse in the left figure. Each of the circles represents the average accuracy at a grating contrast. Only the lowest six tested contrast levels are shown. Accuracy at each contrast tested was averaged and a sigmoidal function was fit to the data. The point at which the curve intersected 70% accuracy is adopted as the grating contrast threshold. CS is the reciprocal of the contrast threshold. **(B)** Left figure, the SF of the grating (gray bars) gradually increased during the testing phase. When the average accuracy (solid line) fell below 70% (dotted line), the SF was decreased gradually and previous SFs were retested repeatedly. Right figure, a frequency-of-seeing curve for the mouse in the left figure. Each of the circles represents the average accuracy at a SF. Only the highest eight SFs tested are presented. A curve was fit to the data using a sigmoidal function, and the intersection with 70% accuracy was adopted as the SF threshold, which was the VA of the mouse.

**FIGURE S2 | Training did not induce physiological changes in mice.** **(A)** Measurement of mouse body weight. Mice in the control (black) and NCT (blue) groups received long-term training beginning at 8 weeks postnatal (pre-training) and ending at 14–15 weeks postnatal (post-training). Mice in both of the groups showed similar gains in body weight compared to the naïve mice (gray). **(B)** The average swim speed of mice in the control and NCT groups before and after training. **(C)** The effects of training on sucrose preference in the naïve, control and NCT groups. Data are expressed as means ± SEM. The numbers in the columns represent the number of animals. Data are expressed as means ± SEM.

**FIGURE S3 | The average learning curves for mice trained with pattern discrimination and visual detection tasks.** Four groups of mice received NSFT training in the V vs. H (*n* = 22), Gaussian (*n* = 18), V vs. G (*n* = 24) and H vs. G (*n* = 8) tasks for 35 consecutive days. The VA was normalized to the individual pre-training value. All of the groups showed significant improvements in VA across training days. Data are expressed as means ± SEM.

**FIGURE S4 | Perceptual training does not change the cortical retinotopic maps of mice.** **(A)** Visual stimuli that were used to measure cortical retinotopy. Elevation and azimuth maps were obtained by stimulating the mouse with moving bars. A color code was used to represent positions of different elevation (top) and azimuth (bottom) lines on the stimulus monitor, with grayscale representing the response amplitude as a fractional change in reflection × 10^4^. The scale bar is 1 mm and applies to all of the panels showing maps. **(B)** Representative elevation **(**top**)** and azimuth (bottom) maps of the control and NSFT mice are displayed. Scale bar, 1 μm. **(C)** Quantification of map scatter (left) and response amplitude (right) for the control (*n* = 9) and NSFT mice (*n* = 10). No significant change was observed in the map scatter (*t*-test, *p* = 0.17 for elevation; *p* = 0.83 for azimuth) or response amplitude (*t*-test, *p* = 0.75 for elevation; *p* = 0.34 for azimuth) between the two groups of mice. Data are expressed as means ± SEM.

## Abbreviations

CS: contrast sensitivity
Gaussian: a Gaussian-ramp masked V vs. H task
H vs. G: a full-screen horizontal sinusoidal grating vs. homogeneous gray
NCT: near the individual contrast threshold
NSFT: near the individual spatial frequency threshold
SF: spatial frequency
SRP: stimulus-selective response potentiation
VA: visual acuity
VPL: visual perceptual learning
V vs. G: a full-screen vertical sinusoidal grating vs. homogeneous gray
V vs. H: a full-screen vertical sinusoidal grating vs. a full-screen horizontal sinusoidal grating
V1: primary visual cortex
