# Supplementary figures and images for: A Mouse Model of Visual Perceptual Learning Reveals Alterations in Neuronal Coding and Dendritic Spine Density in the Visual Cortex
Source: Front Behav Neurosci. 2016 Mar 10;10:42. doi: 10.3389/fnbeh.2016.00042 (PMC4785181; doi:10.3389/fnbeh.2016.00042)

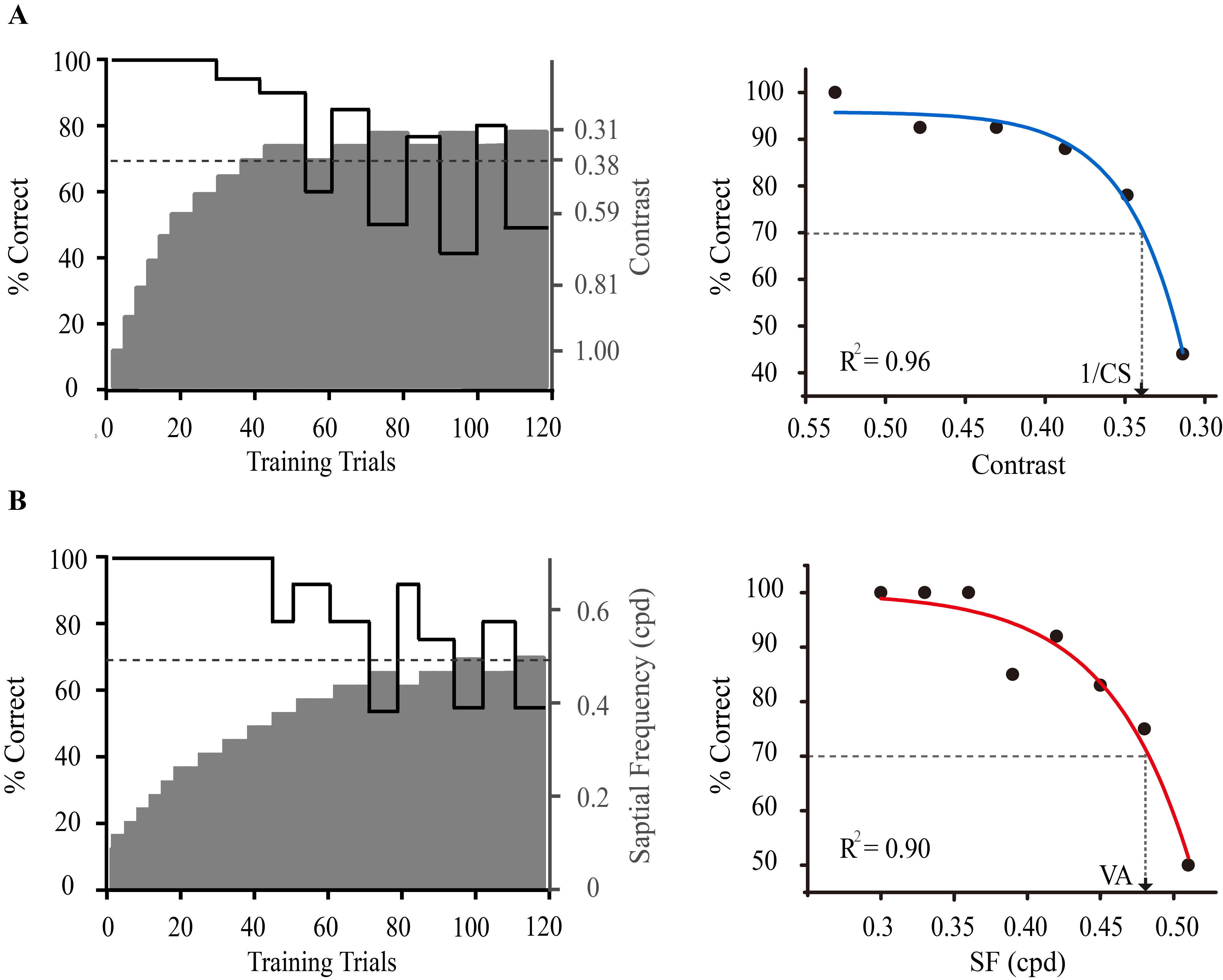

Supplement: Supplementary file 1 [file Image_1.JPEG]

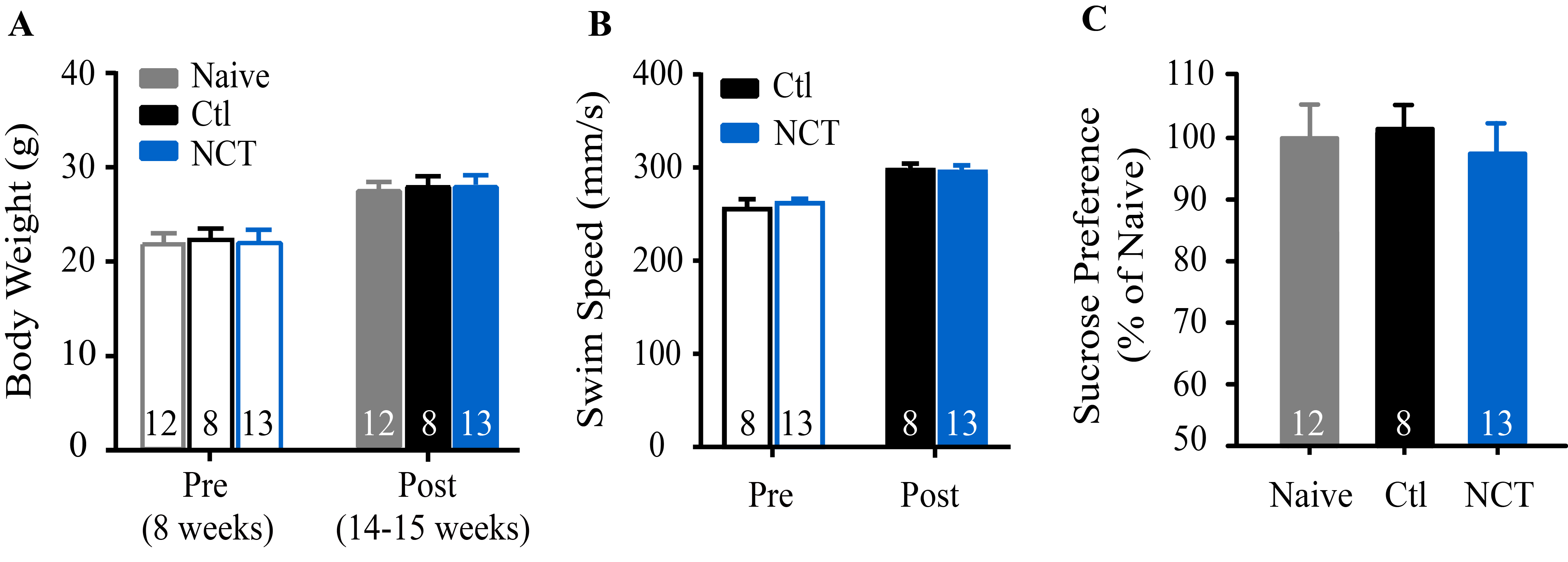

Supplement: Supplementary file 2 [file Image_2.JPEG]

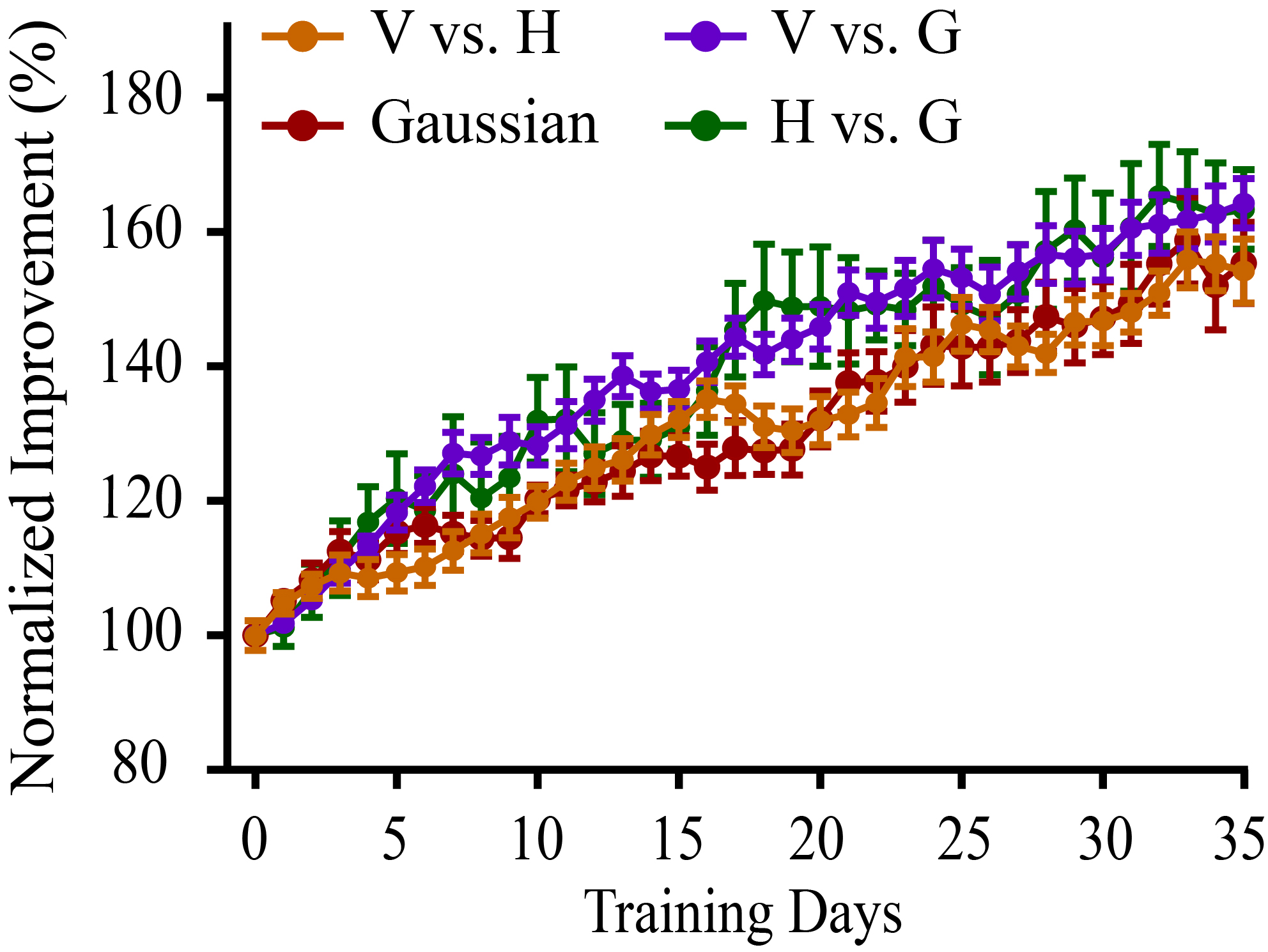

Supplement: Supplementary file 3 [file Image_3.JPEG]

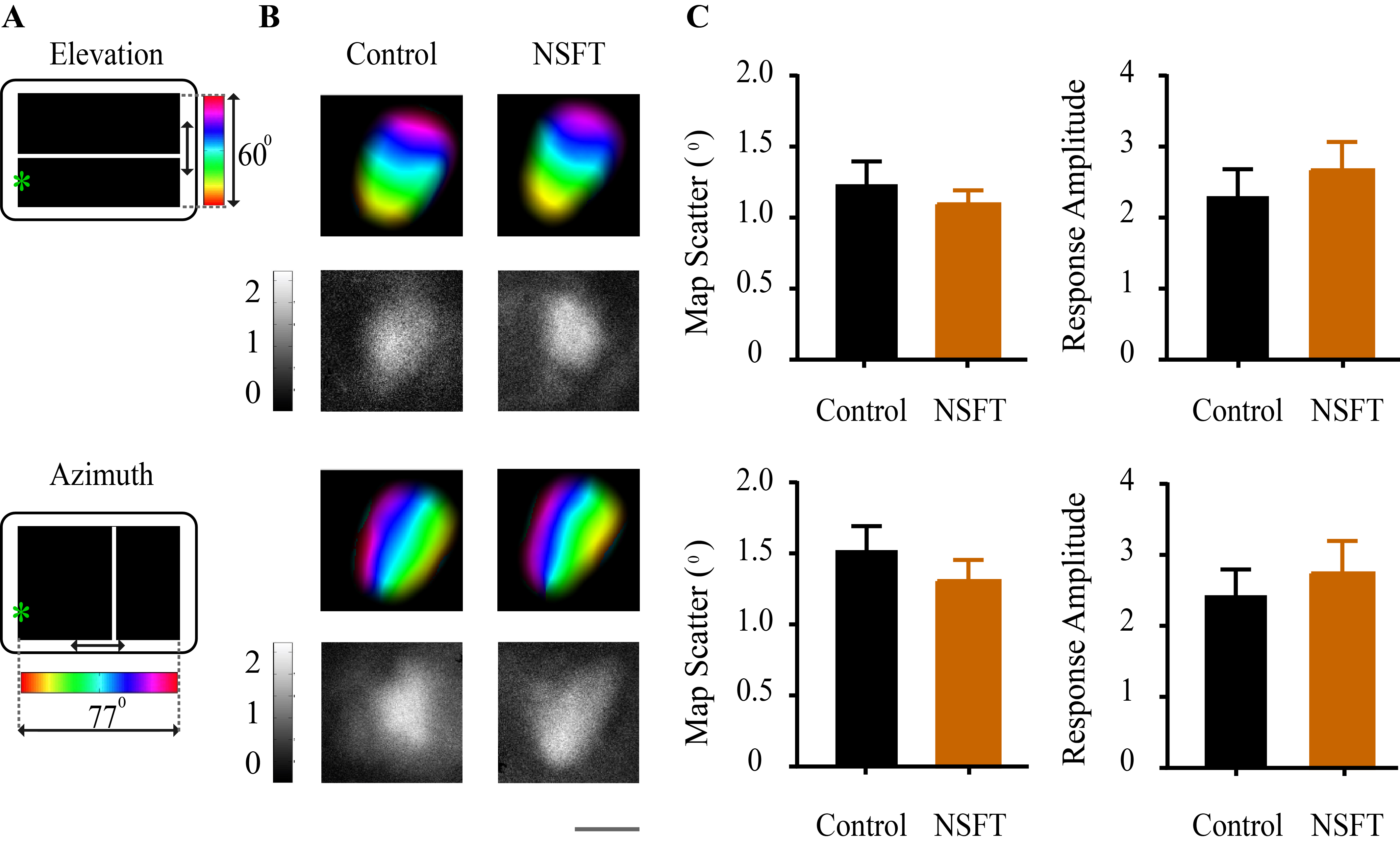

Supplement: Supplementary file 4 [file Image_4.JPEG]
